# *In vitro* effects of selective serotonin reuptake inhibitors on *Cryptococcus gattii* capsule and biofilm

**DOI:** 10.1093/femspd/ftae001

**Published:** 2024-01-10

**Authors:** Letícia Rampazzo da Gama Viveiro, Amanda Rodrigues Rehem, Evelyn Luzia De Souza Santos, Paulo Henrique Fonseca do Carmo, Juliana Campos Junqueira, Liliana Scorzoni

**Affiliations:** Department of Biosciences and Oral Diagnosis, Institute of Science and Technology, São Paulo State University (UNESP), São Paulo 12245-000, Brazil; Department of Biosciences and Oral Diagnosis, Institute of Science and Technology, São Paulo State University (UNESP), São Paulo 12245-000, Brazil; Department of Biosciences and Oral Diagnosis, Institute of Science and Technology, São Paulo State University (UNESP), São Paulo 12245-000, Brazil; Department of Biosciences and Oral Diagnosis, Institute of Science and Technology, São Paulo State University (UNESP), São Paulo 12245-000, Brazil; Department of Biosciences and Oral Diagnosis, Institute of Science and Technology, São Paulo State University (UNESP), São Paulo 12245-000, Brazil; Department of Biosciences and Oral Diagnosis, Institute of Science and Technology, São Paulo State University (UNESP), São Paulo 12245-000, Brazil; Programa de Pós-Graduação em Enfermagem, Universidade de Guarulhos, Guarulhos, São Paulo 07023-070, Brazil

**Keywords:** *Cryptococcus gattii*, Biofilm, Antifungal, Serotonin reuptake inhibitors

## Abstract

Infections caused by *Cryptococcus gattii* mainly affect immunocompetent individuals and the treatment presents important limitations. This study aimed to validate the efficacy of selective serotonin reuptake inhibitors (SSRI), fluoxetine hydrochloride (FLH), and paroxetine hydrochloride (PAH) *in vitro* against *C. gattii*. The antifungal activity of SSRI using the microdilution method revealed a minimal inhibitory concentration (MIC) of 31.25 µg/ml. The combination of FLH or PAH with amphotericin B (AmB) was analyzed using the checkerboard assay and the synergistic effect of SSRI in combination with AmB was able to reduce the SSRI or AmB MIC values 4–8-fold. When examining the effect of SSRI on the induced capsules, we observed that FLH and PAH significantly decreased the size of *C. gattii* capsules. In addition, the effects of FLH and PAH were evaluated in biofilm biomass and viability. The SSRI were able to reduce biofilm biomass and biofilm viability. In conclusion, our results indicate the use of FLH and PAH exhibited *in vitro* anticryptococcal activity, representing a possible future alternative for the cryptococcosis treatment.

## Teaser

Selective serotonin reuptake inhibitors (SSRIs) were active against *Cryptococcus gattii* and in combination with amphotericin B (AmB) exhibited a synergistic effect. Selective serotonin reuptake inhibitors (SSRIs) also reduced capsule size and biofilm, suggesting their potential in cryptococcosis treatments.

## Introduction

According to the Global Action Fund for Fungal Infections (GAFFI), mycoses globally affect >300 million people, and ∼25 million are at serious risk of death (Rodrigues and Nosanchuk 2020). *Aspergillus* spp., *Candida* spp., *Cryptococcus* spp., and *Paracoccidioides* spp. are the main ones responsible for invasive fungal diseases (Gow et al. 2022).

*Cryptococcus gattii*, an emerging fungal pathogen, has demonstrated a notable potential to cause infections in immunocompetent individuals (Springer and Chaturvedi 2010). The treatment of cryptococcosis involves the antifungals amphotericin B (AmB) and fluconazole (Flz). Amphotericin B (AmB), introduced in the late 1950s, has long been considered the gold standard for severe cases (Saag et al. 2000). Fluconazole, a triazole antifungal agent, is used for both induction and maintenance therapy (Perfect et al. 2010). Conventional treatments for cryptococcosis face challenges such as fungal resistance development, notable host toxicity, and limited central nervous system (CNS) penetration for certain medications (Zhai et al. 2012). Consequently, there is a pressing need for the development of alternatives to address systemic mycoses treatment.

Fluoxetine hydrochloride (FLH) and paroxetine hydrochloride (PAH), both from the class of selective serotonin reuptake inhibitors (SSRI) drugs, have received approval as antidepressants for the management of depression and anxiety disorders (Cruz et al. 2020, Murphy et al. 2021). The antifungal potential of SSRI against *Cryptococcus neoformans* has recently been demonstrated at fungicidal concentrations below 10 µg/ml (Pereira et al. 2021) and further, these drugs can act synergistically or additively with Flz *in vivo*, reducing the fungal burden in the brain, kidney, and spleen (Zhai et al. 2012). In this study, we aimed to investigate *in vitro* and biofilm antifungal effects of FLH and PAH against *C. gattii*.

## Materials and methods

### Strains and growth conditions

*Cryptococcus gattii* ATCC 56990 and *C. gattii* clinical isolate 5 (*C. gattii 5*) obtained from the collection from the Oral Microbiology and Immunology Laboratory of the Institute of Science and Technology of São José dos Campos/UNESP were used. Both strains were maintained in Sabouraud dextrose agar (SDA; Difco Laboratories, Detroit, MI, USA) at 37°C. For the assays, yeasts were grown in Sabouraud broth (Difco Laboratories) incubated at 37°C (150 rpm) for 48 h.

### Drugs and antifungals

The SSRI drugs FLH (Fagron, Bologna, Italy), PAH (Infinity Pharma, Hong Kong, China), and the antifungal AmB (Sigma–Aldrich, Saint Louis, MO, USA) were used in this study. A fresh solution of each drug was prepared before each assay using a maximum concentration of 1% dimethyl sulfoxide (Sigma–Aldrich) as a co-solvent to maximize solubility. The intermediate solution was prepared in RPMI 1640 medium (Sigma–Aldrich) for the assays.

### Antimicrobial susceptibility testing

To evaluate the antifungal activity of the SSRI drugs the broth microdilution assay was performed according to the European Committee on Antimicrobial Susceptibility Testing (EUCAST) (Arendrup et al. 2017). Fluoxetine hydrochloride (FLH) and PAH at concentrations from 1.95 to 1000 µg/ml, and AmB at concentrations from 0.03 to 8 µg/ml were prepared. Then, C*. gattii* (2.5 × 10^5^ cells/ml) were added to 96-well plates containing the drugs for treatment and incubated for 48 h for 37ºC. Spectrophotometric readings at 530 nm were performed and the minimal inhibitory concentration (MIC) was defined as the lowest drug concentration that did not allow fungal growth. The minimal fungicidal concentration (MFC) was determined by transferring an inoculum from the wells of microdilution assay to SDA plates using sterile wood picks and further incubation at 37ºC for 48 h. The MFC was defined as the lowest drug concentration that did not permit visual growth of any fungal colonies.

### Evaluation of synergistic effects of the SSRI drugs with AmB

The synergistic effects of SSRI drugs and AmB were assessed using the checkerboard assay, based on the broth microdilution method as described in previous studies (Odds 2003, Arendrup et al. 2017). Concentrations of FLH and PAH ranging from 0.97 to 500 µg/ml, and AmB at concentrations ranging from 0.06 to 8 µg/ml were used. *Cryptococcus gattii* suspensions at 5 × 10^5^ cells/ml were used and subsequently, the plates were incubated at 37ºC for 48 h. Spectrophotometric readings at 530 nm were conducted. To assess synergistic activity, the fractional inhibitory concentration index (FICI) was determined using the formula: ΣFIC = FIC_A_ + FIC_B_, where FIC represents the ratio of the MIC of the drug in combination with the MIC when used alone. Next, FICI was categorized as follows: “synergistic effect” if FICI is ≤ 0.5 and “indifferent” if FICI is > 1 and ≤ 4, and “antagonistic“ relationship was defined as FIC index > 4.0 (Odds 2003).

### Analyses of capsule size

*Cryptococcus gattii* capsules were induced, according to Zaragoza and Casadevall (2004). For this, *C. gattii* at 5 × 10^6^ cells/ml was inoculated in the induction medium (10% Sabouraud broth in 50 mM MOPS, pH 7.4) and incubated for 24 h at 30ºC. Subsequently, cells with induced capsules were removed from the induction medium and treated with subinhibitory drug concentrations (sub-MIC: FLH and PAH = 15.6 µg/ml and AmB = 0.25 µg/ml) in RPMI. The untreated cells were used as the control. The treatment was performed for 24 h at 37ºC. Cells were stained with India ink and analyzed with optical microscopy Axioplan 2 (Zeiss, Germany). Capsule size was measured using the ImageJ software (Rueden et al. 2016) by calculating the difference between the whole cell and the cell body size.

### Biofilm formation and treatment

Biofilm formation was performed as described by Martinez and Casadevall (2006). For biofilm preadhesion, 100 µL of 10^8^ cells/ml of *C. gattii* prepared in RPMI medium supplemented with 2% glucose was added to 96-well plates and incubated at 37ºC without agitation for 24 h. The wells were washed with phosphate-buffered saline (PBS) to remove nonadherent yeasts. Subsequently, 100 µL of the medium was added to the wells and the plate was incubated at 37ºC for 48 h without agitation. After this, wells were washed with PBS to remove nonadherent yeasts. Adherent fungal cells were considered mature biofilms. To evaluate the susceptibility of *C. gattii* biofilms to FLH and PAH, biofilms were treated with 10x of SSRI drugs MIC (312.5 µg/ml), 20 × SSRI drugs MIC (625 µg/ml), or AmB 10x MIC (5 µg/ml) and incubated at 37ºC for 24 h.

### Effect of SSRI drug treatment on *C. gattii* biofilms biomass

Effects of SSRI drug treatment on *C. gattii* biofilms biomass were analyzed using the crystal violet method according to Peeters et al. (2008). After treatments, wells with biofilms were washed with PBS and fixed with absolute ethanol for 15 min. Next, 100 µL of 0.5% crystal violet was added to each well and incubated for 20 min. The excess dye was removed and the wells were washed with PBS. Further, 100 µL of absolute ethanol was added to dilute the dye. The absorbance was measured at 570 nm and the results were expressed as a percentage reduction. *Cryptococcus gattii* biofilms formed after 48 h without treatment were used as the control and the corresponding absorbance values were representative of 100% biomass.

### Effect of SSRI drug treatment on biofilm cell viability

Biofilms were formed as described above, and the effect of SSRI drugs on *C. gattii* biofilm viability was measured by tetrazolium salt (XTT) assay (Martinez and Casadevall 2006). After biofilm formation and drug treatment, wells were washed with 100 µL of PBS and subsequently inoculated with a solution of 50 µL of 1 mg/ml XTT (Sigma-Aldrich) and 4 µL of 1 mM menadione (Sigma–Aldrich). After 1 h of incubation in the dark at 37°C, 50 µL of solution was transferred from each well to another plate and its absorbance was recorded at 490 nm.

### Statistical analyses

Statistical analyses were performed using the GraphPad Prism 5.0 software (GraphPad Software Inc., La Jolla, CA, USA). The data obtained were analyzed using Kruskal-Wallis and Dunn´s. For all tests, the significance level adopted was *P* < 0.05.

## Results

### Susceptibility of *C. gattii* to SSRI drugs and synergistic effect with AmB

Fluoxetine hydrochloride (FLH) and PAH were active against *C. gattii* ATCC 56990 and *C. gattii* 5 with a MIC value of 31.25 µg/ml. Notably, the MFC for both drugs corresponded to the MIC. For *C. gattii* ATCC 56990, FLH + AmB combination resulted in two synergistic concentrations (FLH 3.9 µg/ml + AmB 0.125 µg/ml and FLH 7.8 µg/ml + AmB 0.125 µg/ml) with a 4-fold to 8-fold reduction in MIC values. Additionally, one synergistic combination for PAH + AmB (PAH 7.8 µg/ml + AmB 0.125 µg/ml) with a 4-fold reduction in MIC value was observed when combined with SSRI drugs (Table 1). For the *C. gattii* 5, an indifferent result to the combinations was observed.

**Table 1.** Antifungal susceptibility assay and combinatory effect of SSRI and AmB against *C. gattii*.

Susceptibility test (µg/mL)			Chequerboard assay		MIC reduction	
Drug	MIC	MFC	MICco µg/ml (SSRI + AmB)	FICI (effect)	SSRI drug	AmB
FLH	31.25	31.25	3.9 + 0.125	0.3746 (Syn)	8x	4x
			7.8 + 0.125	0.4996 (Syn)	4x	4x
PAH	31.25	31.25	7.8 + 0.125	0.4996 (Syn)	4x	4x
AmB	0.5	0.5				

The checkerboard assay realized with *C. neoformans* ATCC 90112. MIC, minimal inhibitory concentration; MFC, minimal fungicidal concentration; FICI, fractional inhibitory concentration index; syn, synergistic; MICco, MIC in the combination; SSRIs, selective serotonin reuptake inhibitors; AmB; Amphotericin B.

### Effects of SSRI drugs on *C. gattii* capsule

**Figure 1.**
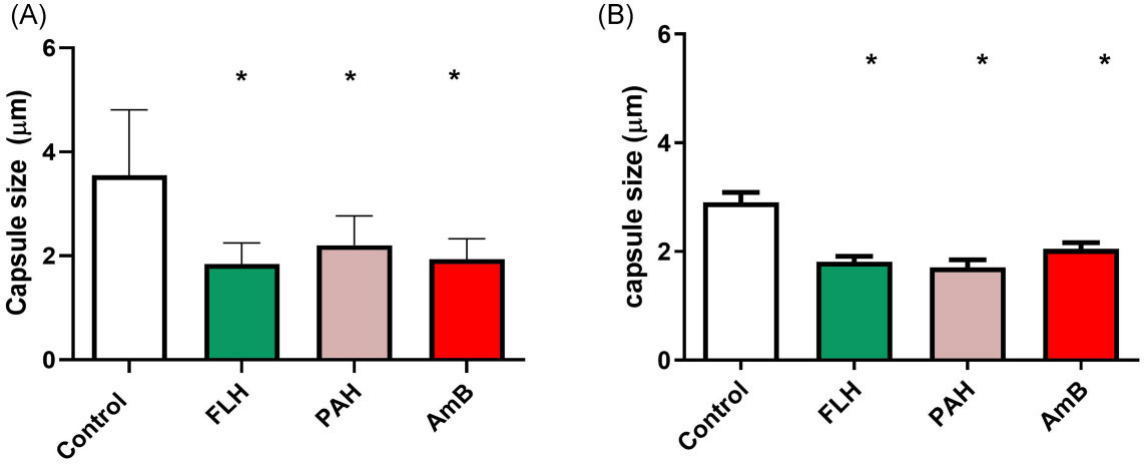
Effect of SSRI treatment on capsule size of *C. gattii* at subinhibitory concentration. *C. gattii* ATCC 56990 (A). *C. gattii* 5 (B) FLH, fluoxetine hydrochloride; PAH, paroxetine hydrochloride; AmB, Amphotericin B. (*): represents statistical difference in relation to the control (p < 0.05).

The sub-MIC of FLH and PAH (15.62 µg/ml) significantly reduced *C. gattii* ATCC 56990 capsule size by 48.16% (*P* < 0.0001) and 38% (*P* < 0.0001), respectively. While AmB (0.25 µg/ml) reduced 45.52% (*P* < 0.0001) the capsule size. For *C. gattii* 5, FLH and PAH (15.62 µg/ml) reduced capsule size by 37.6% (*P* < 0.0001) and 41% (*P* < 0.0001), respectively. While the antifungal drug AmB (0.25 µg/ml) reduced 29.4% (*P* = 0.0003) the capsule size (Fig. 1).

### Effects of SSRI drugs on biofilm biomass

**Figure 2.**
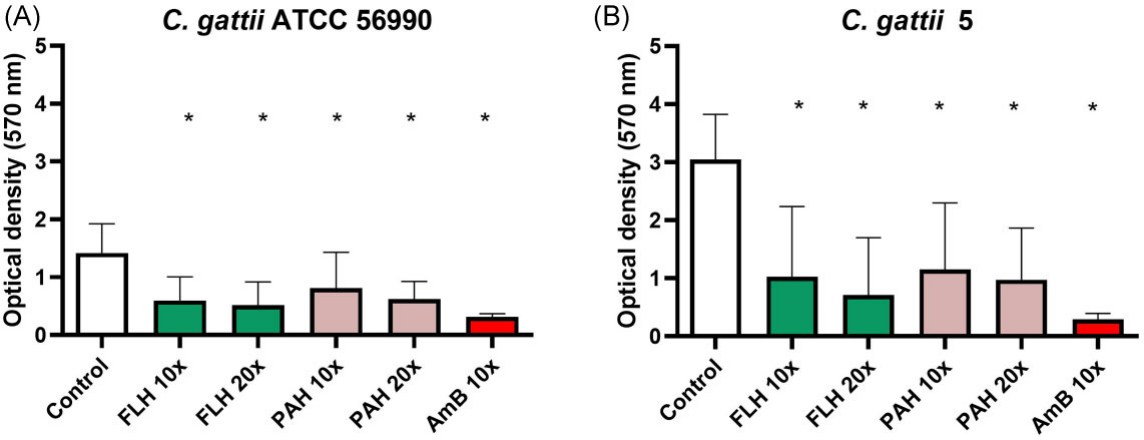
Effect of SSRI treatment on biofilm biomass of *C. gattii. C. gattii* ATCC 56990 (A). *C. gattii 5* (B) FLH, fluoxetine hydrochloride; PAH, paroxetine hydrochloride; AmB, Amphotericin B. 10X, 10 folds the minimal inhibitory concentration; 20X, 20 folds the minimal inhibitory concentration. (*): represents statistical difference in relation to the control (p < 0.05).

In the *C. gattii* ATCC 56990 biofilm, FLH treatment at 10x MIC and 20x MIC reduced biofilm biomass by 57.72% (*P* = 0.0002) and 63.44% (*P* < 0.0001), respectively. Paroxetine hydrochloride (PAH) at 10x MIC and 20x MIC decreased biofilm biomass by 42.69% (*P* = 0.0091) and 56.03% (*P* = 0.0009), respectively. Amphotericin B (AmB) at 10x MIC, used as a control, reduced biofilm biomass by 77.98% (*P* < 0.0001) (Fig. 2A).

**Figure 3.**
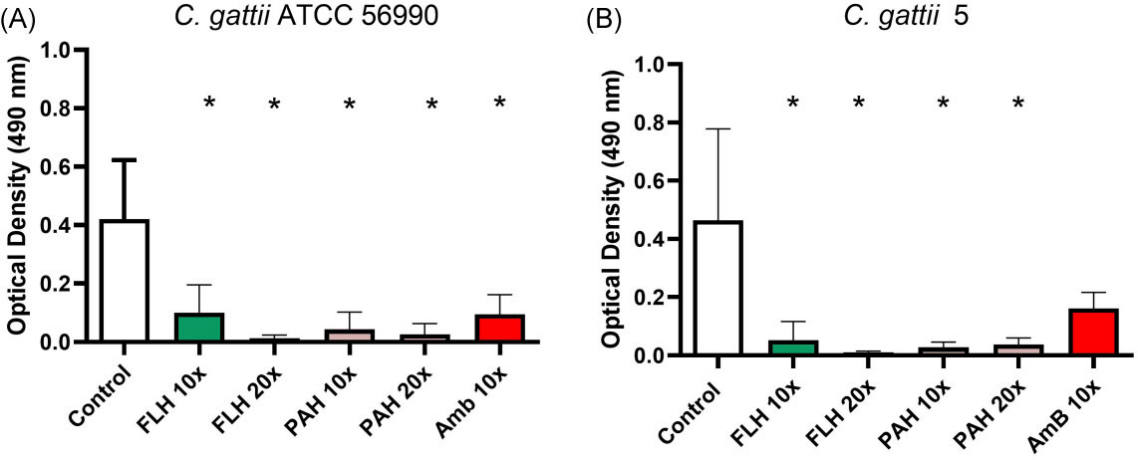
Effect of SSRI treatment on the biofilm viability of *C. gattii. C. gattii* ATCC 56990 (A). *C. gattii* 5 (B) FLH, fluoxetine hydrochloride; PAH, paroxetine hydrochloride; AmB, Amphotericin B. 10X, 10 folds the minimal inhibitory concentration; 20X, 20 folds the minimal inhibitory concentration. (*): represents statistical difference in relation to the control (p < 0.05).

For *C. gattii* 5, the treatment with FLH reduced biofilm biomass in 66.34% (*P* < 0.0001) at 10x MIC and in 76.66% (*P* < 0.0001) at 20x MIC (Fig. 3D). The treatment with PAH at 10x MIC and 20x MIC decreased biofilm biomass by 62.17% (*P* = 0.0005) and 68.03% (*P* = 0.0003) respectively. AmB at 10x MIC, used as a control, reduced 90.40% (*P* < 0.0001) of biofilm biomass (Fig. 2B).

### Effects of SSRI drugs on biofilm viability

The effect of the SSRI drugs on *C. gattii* biofilms was evaluated at 10x and 20x MIC and AmB at 10x MIC. For *C. gattii* ATCC 56990 the treatment with FLH revealed a decreased biofilm viability by 39.05% (*P* = 0.0109) and 78.94% (*P* = 0.0082) at 10x MIC and 20 × MIC, respectively. The biofilm viability percentage with treatment with PAH was 56.99% (*P* < 0.0001) and 67.64% (*P* < 0.0001) for 10x MIC and 20 × MIC, respectively. AmB 10x MIC used as a control, decreased biofilm viability by 42.24% (*P* = 0.0037) (Fig. 3A).

For *C. gattii* 5, the treatment with FLH revealed a decreased biofilm viability by 57.13% (*P* < 0.0001) and 84.62% (*P* < 0.0001) at 10x MIC and 20 × MIC, respectively. The biofilm viability percentage decreased for treatment with PAH was 65% (*P* < 0.0001) and 58.64% (*P* < 0.0001), for 10x MIC and 20× MIC, respectively. AmB 10x MIC, used as a control, decreased biofilm viability by 20.83% (*P* > 0.9999). All concentrations of both SSRI drugs exhibited statistically significant differences compared to the control group (Fig. 3B).

## Discussion

Fungal infections have shown a significant increase in recent years, with cryptococcosis being notably prevalent among both immunocompromized and immunocompetent individuals (Perfect et al. 2010, Spitzer et al. 2017). Resistance to conventional treatments has been reported for *Cryptococcus* spp., coupled with the scarcity of antifungal options, elevated costs, and significant host toxicity, highlighting the imperative need for treatment alternatives (Rodero et al. 2003, Scorzoni et al. 2017).

Drug repositioning is a promising alternative to conventional antifungal treatment (Katragkou et al. 2016, Pushpakom et al. 2019) and drugs from the SSRI class have shown activity against fungal agents, including *Candida* spp, *Aspergillus* spp. and *C. neoformans* (Lass-Florl 2001, Oliveira et al. 2014, Pereira et al. 2021). However, there are no reports of SSRI drugs activities on *C. gattii*.

SSRI drugs have a proven inhibitory effect on *Candida* spp. by reducing biofilm formation and causing cell death by apoptosis. In addition, FLH was shown to inhibit fungal growth at concentrations of 127-508 µg/ml, and PAH at concentrations of 80–320 µg/ml (Costa Silva et al. 2017, Oliveira et al. 2018). For *C.neoformans*, the concentrations able to inhibit fungal growth were 9.6 µg/ml for FLH and 41 µg/mLlfor PAH (Pereira et al. 2021). In this study, both drugs, FLH and PAH showed a MIC of 31.25 µg/ml for *C. gattii* ATCC 56990 and *C. gattii 5*. When combined effects of SSRI drugs with AmB were evaluated, three synergistic combinations were found for *C. gattii* ATCC 56990, which reduced the MIC values by 4–8-fold.

*Cryptococcus* spp. capsule is a virulence mechanism essential for the pathogenicity of this yeast. It hinders phagocytosis and modulates host responses, contributing to disease progression. Understanding the capsule's molecular mechanisms is essential for developing effective cryptococcosis treatments (Zaragoza et al. 2008, Vecchiarelli et al. 2013). In this study, we demonstrated that subinhibitory concentration of FLH and PAH (15.62 µg/ml) reduced capsule size by up to 37.6%. In *C. neoformans*, at sub-MIC concentrations, FLH (4.8 µg/ml) and PAH (20 µg/ml) markedly decreased capsule size by up to 63% (Pereira et al. 2021).

Therefore, we evaluated the antibiofilm activity of the SSRI, FLH, and PAH on *C. gattii*. Our results indicate a significant reduction of biofilm biomass and viability by both drugs at 10x and 20x MIC. Studies with drugs from other classes, such as the anthelmintic benzimidazoles, have revealed a reduction in biofilms against *C. neoformans*, also proving active for *C. gattii* (Joffe et al. 2017).

The mechanisms action of the effect of SSRI on *C. gattii* is poorly described. Sertraline has been shown to affect intracellular membrane organization, translation, and vesicle transport (Zhai et al. 2012), given that both drugs are from SSRI, with a comparable mechanism of action, FLH and PAH could be acting similarly.

The treatment of systemic mycoses is considerably complicated by the limited number of antifungal drugs and the poor penetration of antifungals in the CNS due to the blood-brain barrier, only a few fungistatic drugs show reasonable penetration into the CNS. On the other hand, when the concentrations of SSRI drugs such as sertraline were analyzed in the cerebrospinal fluid and brain, they were ∼20–40 times higher compared to plasma levels (Lass-Florl 2001, Marchetti et al. 2004). Sertraline exhibited potent antifungal activity against *C. neoformans*, the main etiological agent of cryptococcal meningitis (Zhai et al. 2012). In addition, sertraline decreased the fungal burden in the brain and spleen of mice using murine model of cryptococcosis (Treviño-Rangel et al. 2016). Additionally, according to the British Pharmacopeia, lethal dose 50 (DL50) of SSRI drugs here evaluated are 374 mg/kg (PAH) and 452 mg/kg (FLH) when administrated orally in rats. Therefore, *in vivo* studies with SSRIs are needed to evaluate the efficiency of these drugs.

In summary, this study provides evidence of the potent anticryptococcal activity of FLH and PAH *in vitro* and its effects on *C. gattii* capsules and biofilm. However, future *in vivo* investigations to better describe these activities are required to demonstrate the relevance of FLH and PAH as antifungal agents for cryptococcosis.
